# Omega 3 polyunsaturated fatty acids inhibit cell proliferation by regulating cell cycle in *fad3b* transgenic mouse embryonic stem cells

**DOI:** 10.1186/s12944-018-0862-x

**Published:** 2018-09-08

**Authors:** Zhuying Wei, Dongfang Li, Lin Zhu, Lei Yang, Chen Chen, Chunling Bai, Guangpeng Li

**Affiliations:** 1 State Key Laboratory of Reproductive Regulation & Breeding of Grassland Livestock, College of Life Sciences, Inner Mongolia University, Hohhot, 010070 China; 2 Inner Mongolia People’s Hospital, Hohhot, 010017 China; 3 College of Life Science, Inner Mongolia University, Hohhot, 010070 China

**Keywords:** Embryonic stem cells, fad3b, Cell cycle, P21, Cdk4

## Abstract

**Background:**

The consumption of omega 3 polyunsaturated fatty acids (PUFAs) is important for human health and is closely associated with cell proliferation and differentiation. This study aimed to investigate the influence of omega 3 PUFAs on embryonic stem cell (ESC) proliferation and explore potential mechanisms that mediate these effects.

**Methods:**

In this study, we isolated ESCs from *fad3b*-expressing transgenic mice. We detected the fatty-acid composition of ESCs using gas chromatography-mass spectroscopy, analyzed cell-cycle phases using flow cytometry, and detected gene expression using real-time polymerase chain reaction (PCR) and western blots.

**Results:**

The amount of omega 3 PUFAs significantly increased in *fad3b* versus control ESCs. However, the growth of *fad3b* ESCs was slower than that of control cells, and most *fad3b* ESCs were in a prolonged G0/G1 phase after being passaged for 18 h. Therefore, we hypothesized that *fad3b* expression inhibited the cell cycle in ESCs by increasing the expression of P21, which then decreased the expression of cyclin-dependent kinase 4 (Cdk4). We found that pretreatment of *fad3b* ESCs with PD0325901, a P21 inhibitor, clearly attenuated the inhibitory effects of P21 on Cdk4, and resumed the cell cycle.

**Conclusions:**

Expression of the *fad3b* gene in ESCs increased the omega 3 PUFA content, which inhibited cell proliferation by prolonging the G1 phase but did not arrest the G0-to-G1 or G1-to-S transitions. The prolonged G1 phase in *fad3b* ESCs was probably induced by downregulation of Cdk4 expression via p21 upregulation. These results suggest that accumulation of omega 3 PUFAs in vivo may beneficially affect ESC differentiation and that *fad3b* ESCs may be a useful tool for investigating related mechanisms.

**Electronic supplementary material:**

The online version of this article (10.1186/s12944-018-0862-x) contains supplementary material, which is available to authorized users.

## Background

Omega 3 polyunsaturated fatty acids (PUFAs) are believed to be closely involved in immune regulation, aging, anti-inflammatory activity, cell proliferation, and cell differentiation [1]. In mammals, omega 3 PUFAs must be sourced from dietary components due to a lack of fatty acid desaturases, which catalyze omega 6 fatty acids into omega 3 PUFAs [2]. However, transgenic expression of fatty acid desaturase genes could provide an alternative pathway for mammals to transform omega 6 to omega 3 PUFAs themselves.

Previous studies primarily used the *Caenorhabditis elegans fat1* gene as a transgenic fatty acid desaturase [3–6]. Fad3b is an endoplasmic reticulum transmembrane protein that functions similarly to Fat1 [7] and is relatively suitable for expression in mammalian cells [8]. The primary omega 3 PUFAs are docosahexanoic acid (DHA) and eicosahexanoic acid (EPA). The mechanism that controls the effect of omega 3 PUFAs on cell-cycle regulation and physiological activity is not well characterized [9]. It is possible that variations in the concentrations of omega 3 PUFAs and in treatment times of the exogenous fatty acids resulted in the inconsistent results observed by different research groups [10]. For example, the addition of DHA to tumor cells arrested in G1 phase increased expression of p21 and decreased expression of cyclin D1 and cyclin E in one study [11], but decreased expression of the Cdk2 and cyclin E proteins and induced apoptosis in another study [12]. In endothelial cells, the addition of 17,18-epoxy-EPA decreased cell proliferation by down-regulating the cyclin D1/cyclin-dependent kinase (Cdk)-4 complex [13]. By contrast, EPA addition to leukemic k-562 cells promoted accumulation of G0/G1 cells and down-regulated cyclin E expression [14]. Interestingly, addition of both DHA and EPA to myoblast cells decreased cell growth and cell accumulation at G1 by decreasing expression of Cdk2 and cyclin E expression [15]. However, DHA addition in neural stem cells promoted cell-cycle progression, inhibited apoptosis, and induced neurogenesis [16].

The cell cycle and proliferation of ESCs is different than that of somatic cells in that ES cells have a short G1 phase and devote about half of their entire cycle to S phase [17]. In most cases, a prolonged G1 phase is associated with differentiation, but artificially extending the G1 phase by knocking down Cdk4/6 or by overexpressing the Cdk inhibitor p21 does not significantly affect ESC pluripotency [18].

In this study, we used a transgenic mouse model expressing the *fad3b* gene from flax (*Linum usitatissimum*) to investigate the mechanism of how endogenously expressed omega 3 PUFAs affect cell-cycle regulation in ESCs. We show that *fad3b* expression in ESCs increased the omega 3 PUFA content, and then induced a prolonged G1 phase by down-regulating Cdk4 expression via p21 upregulation.

## Methods

### Animals

The *fad3b* mice aged 6–8 weeks were obtained from the Research Center for Laboratory Animal Science Inner Mongolia University. All experimental mice were maintained in conventional animal housing with a 12 h light/dark photoperiod and free access to food and water. This study was carried out in strict accordance with the guidelines of Experimental Animal Management and Operation Standard of Inner Mongolia University.

### Isolation and culture of *fad3b* ESCs

The blastocysts were collected at 3.5 days post coitum from the uterus of *fad3b* mice and inoculated onto 24-well plates with mouse embryonic fibroblast feeder cells. After 4–6 d, we selected well-shaped clones, digested these with 0.05% trypsin, and then transferred cells onto a new feeder layer [19]. The cells were cultured over 5–30 generations for subsequent cell identification experiments. To verify the regulatory role of *fad3b* in the cell cycle, we added 1 μM PD0325901 (Sigma, USA) to the culture medium after passage and collected the cells 18 h later.

### Immunohistochemical analysis

The detected cells were washed three times with Dulbecco’s phosphate-buffered saline (DPBS), fixed for 30 min in 4% paraformaldehyde (PFA) at room temperature, then treated with 0.5% Triton X-100 for 10 min. Cells were incubated with primary antibodies against Oct4, Sox2, Nanog, and SSEA-1 (1:100, cell signaling technology, USA) at 4 °C overnight then incubated with fluorescence-tagged secondary antibodies (1:500, Santa Cruz, USA) at 37 °C for 2 h. After incubation, we stained with 4′,6-diamidino-2-phenylindole (DAPI) for 10 min after mounting and then observed and photographed samples under a confocal fluorescence microscope (A1R/A1, Nikon, Japan). For the alkaline phosphatase activity assay, we followed the instructions of the BCIP/NBT Alkaline Phosphatase Color Development Kit (Beyotime, China).

### Teratoma preparation and analysis

The ESCs were digested, removed from feeder cells, and collected at 10^7^ cells per 0.5 ml of phosphate-buffered saline (PBS) [19]. The resuspended ESCs were injected into the groins of nude mice. After 4–5 weeks, we removed the resultant tumors, performed paraffin sectioning and hematoxylin and eosin staining, and examined the sections under the microscope (A1, Carl Zeiss, German).

### Fatty acid methyl ester analysis

The ESCs were collected in 15 ml tubes, washed twice with DPBS, and then 1 ml methanol solution containing 2.5% H_2_SO_4_ was added. Samples were then placed in an 80 °C water bath for 90 min. After cooling to room temperature, we added 1.5 ml of 0.9% NaCl to the tubes, mixed well, vortexed for 5 min, and centrifuged for 5 min at 2000 rpm/min to extract fatty acids into the organic phase. The supernatant was then transferred to new 1.5 ml tubes. We added 0.4 ml saturated KOH methanol solution, mixed well, vortexed for 5 min, and centrifuged for 10 min at 2000 rpm/min. The supernatant was collected in a sample bottle, and we analyzed the fatty-acid content using gas chromatography-mass spectroscopy (GC-MS) (GC-MS 2010 plus, Shimadzu, Japan). The fatty-acid levels are reported as percentage of total content.

### Detection of the cell-cycle phase using flow cytometry

The ESCs were collected in centrifuge tubes, resuspended with propidium iodide (PI) staining solution (10 mg/ml RNase 10 μl, 1 mg/ml PI 50 μl, Triton X-100 2.5 μl, DPBS 940 μl), and mixed gently and evenly. After 15 min, we performed flow cytometry (FACSAria II, BD Company, USA) to detect the cell-cycle phase using a 488 nm laser. At least 1 × 10^4^ cells were recorded. BD FACSDiva software was used to analyze the percentage of cells in G0/G1, S, and G2/M phases based on DNA content [20].

### Calculations of cell growth rate and doubling time

We inoculated 1×10^5^ ESCs into each well of a 6-well plate. After 12 h, we digested the ESCs with 0.05% trypsin and then counted cells. We repeated the digestion and cell count every 6 h until the 96-h endpoint. Then, we drew ESC growth curves and calculated the doubling time of the cells according to the formula: DT = t × [ln2/ (lnNt-lnNo)], where T is the culture time, No is the initial number of cells, and Nt is the number of cells at the time t.

### Real-time PCR

The RNA from ESCs was isolated using the RNAiso Plus kit (Takara 9108) and reverse transcribed into cDNA using an Omniscript RT system (Takara RR047A) and oligo-dT primers. We amplified cDNA using an ABI7500 real-time PCR system (Applied Biosystems, America) and SYBR Green (Takara RR820A). The PCR primers are listed in Additional file 1: Table S1. The protocol for PCR amplifications was as follows: 95 °C for 30 s initially, followed by 40 cycles at 95 °C for 5 s, 60 °C for 34 s, and a final melt curve stage.The cycle threshold (Ct) values of targeted genes were normalized to the housekeeping gene *Gapdh* using the 2^-ΔΔCt^ method [21].

### Western blotting

The collected ESCs were lysed on ice using the Mammalian Protein Extraction Reagent (CWBiotech, China). The protein concentration was determined using Varioskan Flash (Thermo Fisher Scientific, USA). We boiled the lysate supernatant for 10 min, separated samples on a 12% sodium dodecyl sulfate (SDS)-polyacrylamide gel, and transferred proteins to a polyvinylidene fluoride (PVDF) membrane (Millipore, USA). The membrane was blocked in 5% skim milk in Tris-buffered saline with Tween (TBST) for 1 h and then incubated with antibodies against Fad3b (1:50000, ABclonal, China),P21, Cdk4, Cdk6, and α-tubulin (1:500, Abcam, USA) overnight at 4 °C. The membrane was then incubated with donkey peroxidase-conjugated goat anti-rabbit immunoglobulin G (IgG) antibody (diluted 1:5000; Jackson Immunoresearch, USA) for 3 h at room temperature before washing with TBST. Signal was detected using a Tanon-5200 chemiluminescence detector (YuanPingHao Biotech, China).

### Calculations and statistical analyses

The data are expressed as means ± standard error of the mean of at least three independent experimental repeats. In graphs, all bars represent means and error bars represent one standard deviation. The statistical analyses were performed by Welch’s two-tailed t-test when comparing two groups with unequal standard deviations. When comparing multiple groups, a one-way analysis of variance test without replication was performed followed by post hoc analysis using Bonferroni correction to adjust for multiple comparisons. We considered *p < 0.05 and **p < 0.01 to be statistically significant.

## Results

### Establishment of *fad3b* ESCs

**Fig. 1:**
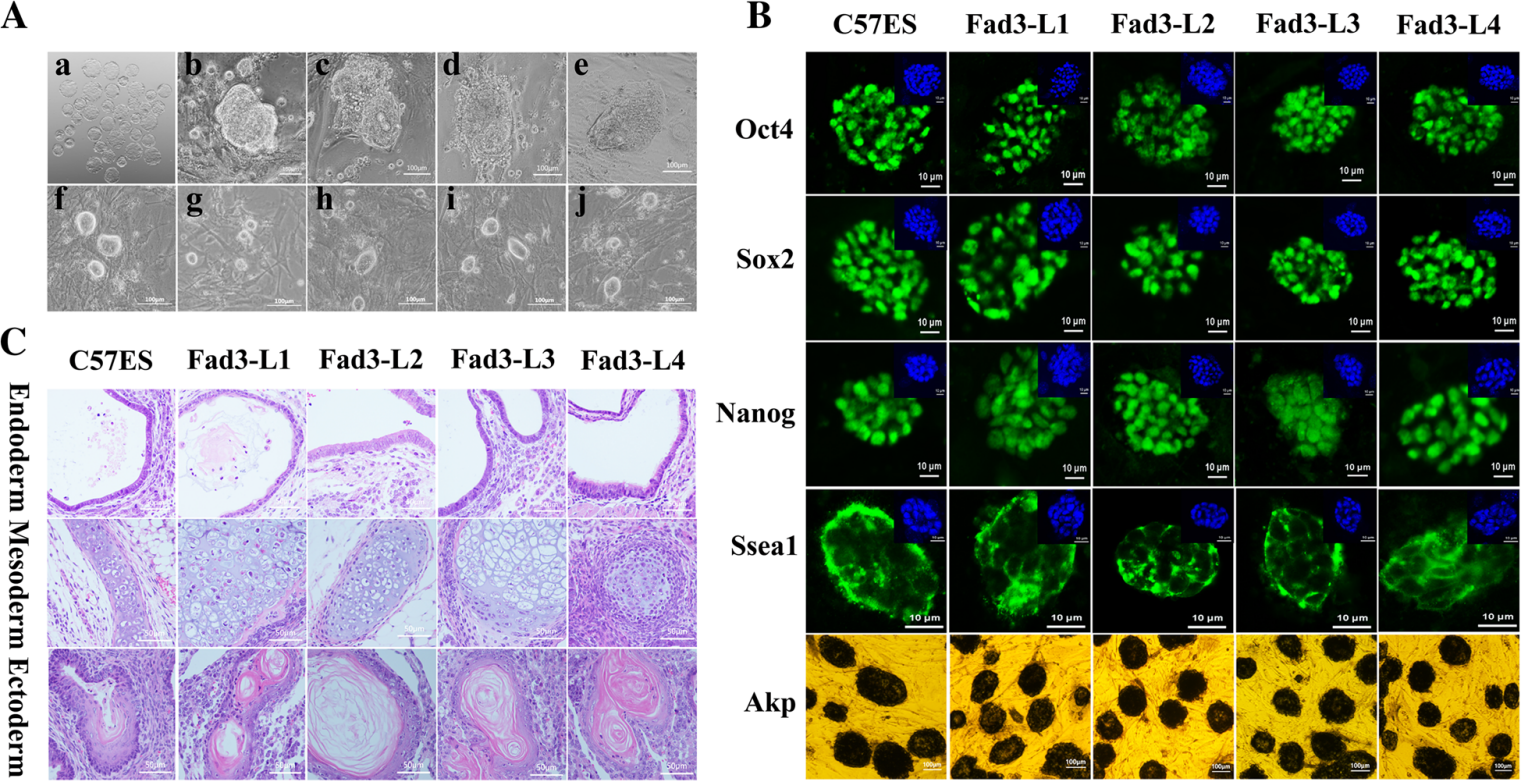
Establishment and identification of *fad3b*-expressing mouse embryonic stem cells (ESCs) lines. **a**. The establishment of *fad3b* mouse ESCs. a: *fad3b* blastocysts; b-e: primary cells grown from *fad3b* blastocysts; f: C57 ESCs; g-h: *fad3b* ESCs. **b**. Expression of Oct4, Sox2, Nanog, Ssea1, and AKP in *fad3b*-expressing mouse ESCs. **c**. Teratoma staining of in vivo germ-layer differentiation in *fad3b* mouse ESCs

We collected a total of 29 blastocysts (Fig. 1aa) with normal morphology from five *fad3b* transgenic mice. From these blastocysts, we obtained four *fad3b* ESC lines and defined these as the Fad3-L1 to L4 lines (Fig. 1ab-e). The lab-kept C57 ESC line was used as a control for subsequent experiments (Fig. 1af). Compared with the control, the *fad3b* ESC lines had characteristics consistent with those of undifferentiated mouse ESCs and had no obvious morphological difference from control cells (Fig. 1ag-h). The *fad3b* ESCs both expressed the key markers of ESCs (Fig. 1b) and were able to differentiate into three germ-layer structures in vivo (Fig. 1c).

### Expression *fad3b* altered fatty-acid metabolism and the composition of ESCs

**Fig. 2:**
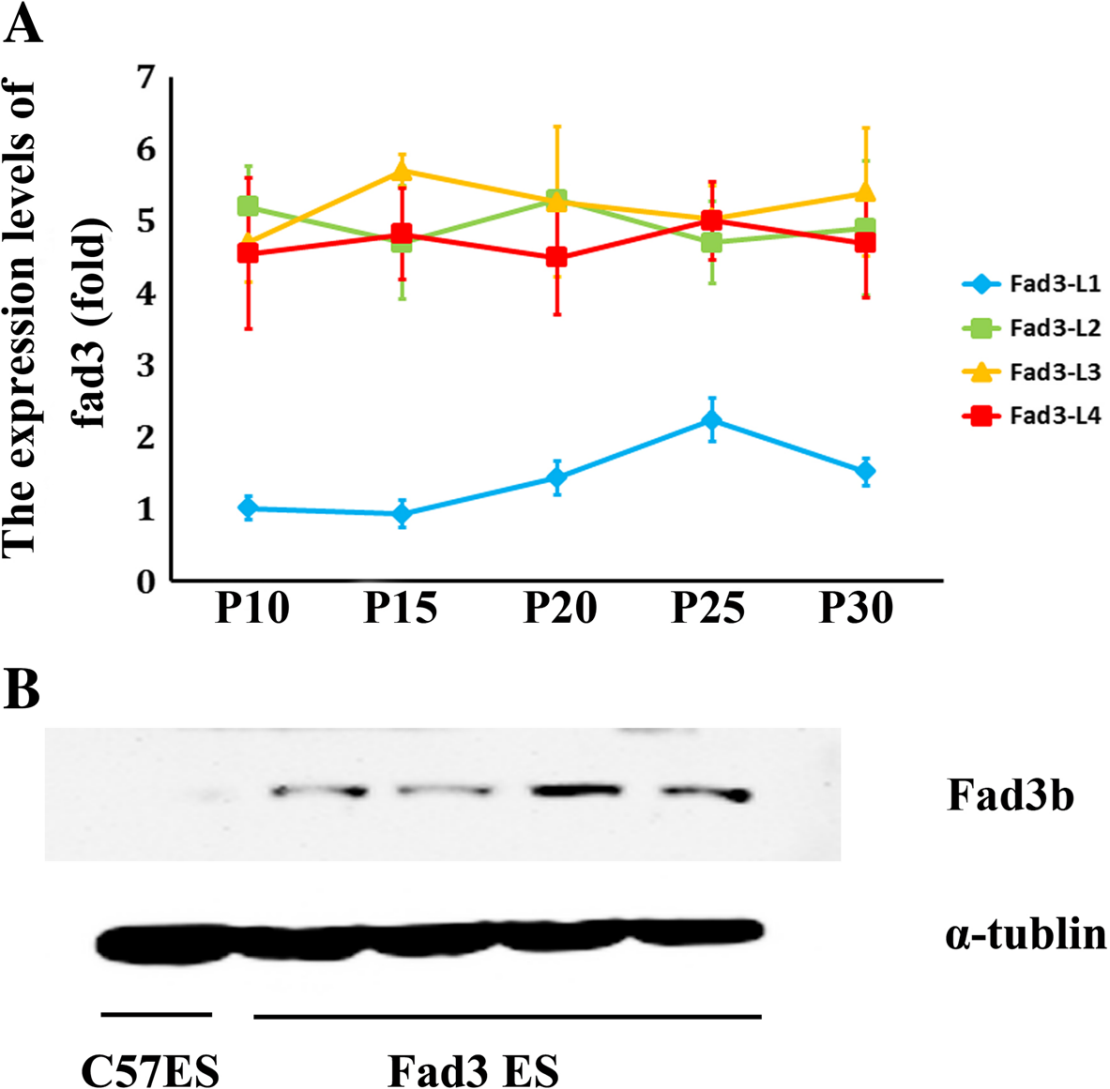
The *fad3b* expressing in ESCs. **a**. Stable *fad3b* mRNA expression in Fad3-L1 to L4 cell lines. **b**. Fad3b protein expression in ESCs

**Fig. 3:**
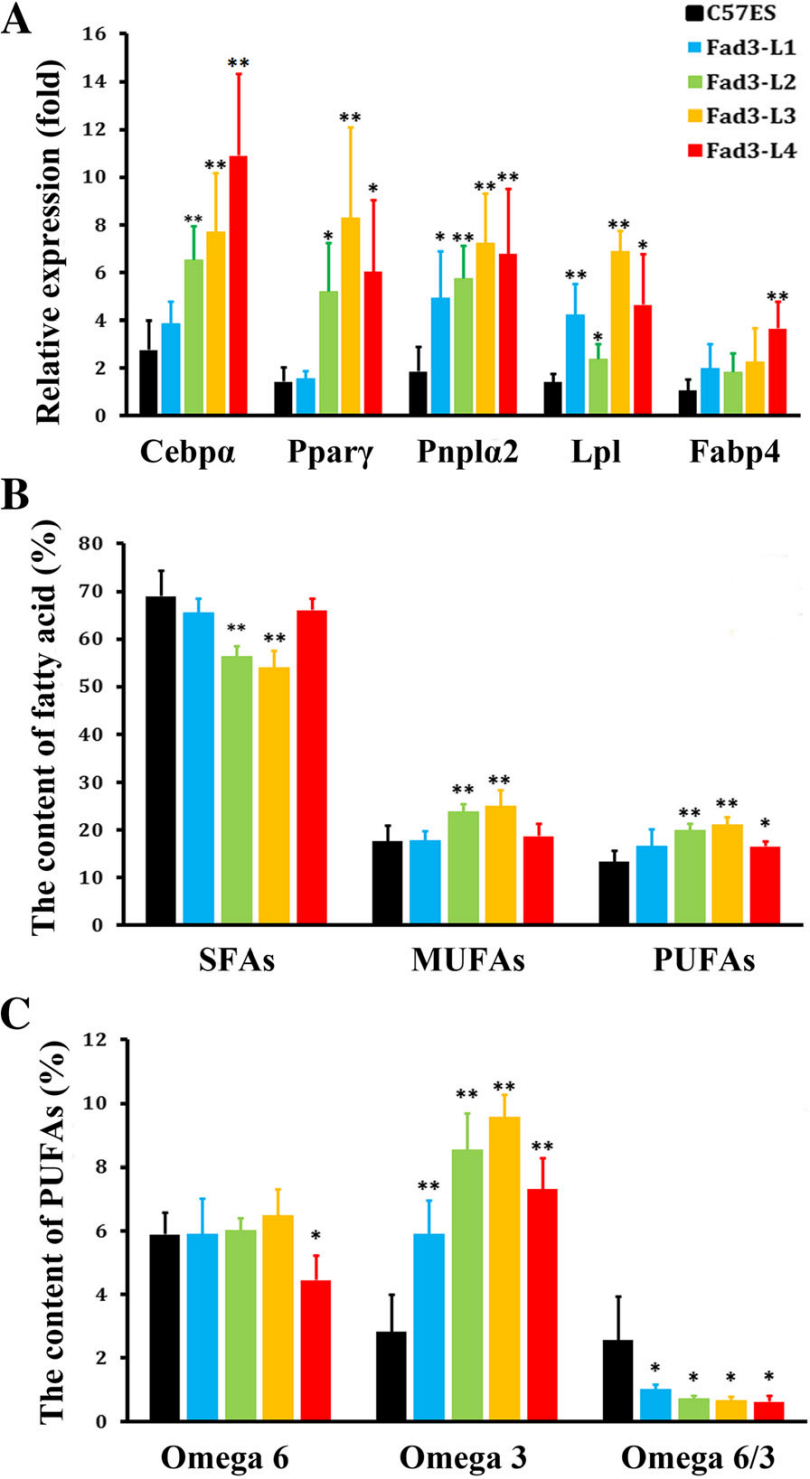
The ESCs expressing *fad3b* had fatty acid desaturase activity. **a**. The expression of lipid metabolism genes in *fad3b* ESCs. **b**. Saturated fatty acid (SFA), monounsaturated fatty acid (MUFA), and polyunsaturated fatty acid (PUFA) content in *fad3b* ESCs and in control cells. **c**. Levels of omega 6 PUFAs and omega 3 PUFAs in *fad3b* ESCs and control cells

The expression of *fad3b* in the Fad3-L1 to L4 cell lines was relatively stable. However, the expression of *fad3b* mRNA was not consistent across the four cell lines, the *fad3b* mRNA expression in Fad3-L1 was less than that in L2, L3, and L4 (Fig. 2a). The *fad3b* protein expression was showed in Fig. 2b. The expression of lipid metabolism-related genes *Lpl*, *Fabp4*, *Pnpla2*, *PPARα*, and *Cebpα* at the 10th to 30th passages in the *fad3b* ESCs were upregulated (Fig. 3a).

The fatty-acid content in the *fad3b* ESCs was changed (Tab.S2), along with the average levels of saturated fatty acids (SFAs), monounsaturated fatty acids (MUFAs), and PUFAs at 10th to 30th passages, as shown in Fig. 3b and c. The SFA content was lower in *fad3b* ESCs, especially in L2 and L3 (56.39 ± 2.03% and 53.93 ± 3.48%, respectively, *p* < 0.01). The MUFAs content was higher in the *fad3b* ESCs, especially L2 and L3 (23.77 ± 1.64% and 25.02 ± 3.30%, respectively, p < 0.01) than in control cells (17.61 ± 3.30%). The PUFA content was higher in the *fad3b* ESCs, especially in L2, L3, and L4 (19.22 ± 1.31%, 21.06 ± 1.69%, and 16.61 ± 1.08%, respectively, p < 0.01or 0.05) than in control cells (13.34 ± 2.21%). The specific analysis of PUFA composition found that the omega 6 PUFAs in the *fad3b* ESCs were almost the same as in controls, except for the L4 line. However, the omega 3 PUFAs content was significantly higher in L1 to L4 cell lines (5.90 ± 1.04%, 8.52 ± 1.16%, 9.55 ± 0.71%, and 7.30 ± 0.97%, respectively, p < 0.01) than in the controls (2.83 ± 1.17%). The ratio of omega 6 to omega 3 fatty acids was significantly lower in L1 to L4 cell lines (1.01 ± 0.16%, 0.72 ± 0.08%, 0.68 ± 0.11%, and 0.64 ± 0.18%, respectively, *p* < 0.05) than in controls (2.56 ± 1.35%). Thus, expression of *fad3b* altered the fatty-acid composition in the ESCs by increasing the proportion of MUFAs and PUFAs, particularly that of omega 3 PUFAs.

### *Fad3b* expression prolonged ESCs in G0/G1 phase by regulating p21 and Cdk4

**Fig. 4:**
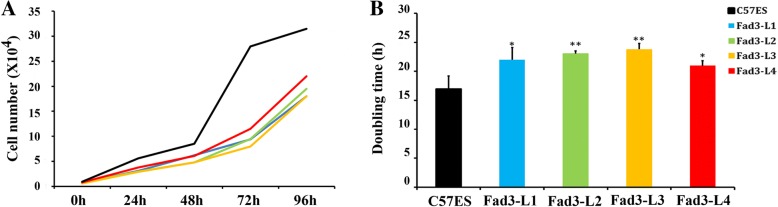
The growth rate of *fad3b* ESCs decreased significantly. **a**. The growth curve of *fad3b* ESCs. **b**. The doubling time of *fad3b* ESCs

The cell-cycle transition of undifferentiated mouse ES cells is dependent on the cell line and cultivation conditions [22]. As showed in Fig. 4, the growth rate of *fad3b* ESCs was slower down than that of the control cells (Fig. 4a). Our calculation of cell doubling times showed that the control cell (C57 ESCs) doubling time was 16.99 ± 2.22 h, whereas the doubling times of Fad3-L1 to L4 cells were 21.98 ± 2.13 h, 23.07 ± 0.46 h, 23.80 ± 0.98 h, and 20.97 ± 0.87 h, respectively (Fig. 4b). The doubling times of the *fad3b* ESCs were significantly longer than that of the controls, suggesting that *fad3b* expression affected cell growth and prolonged the cell cycle.

**Fig. 5:**
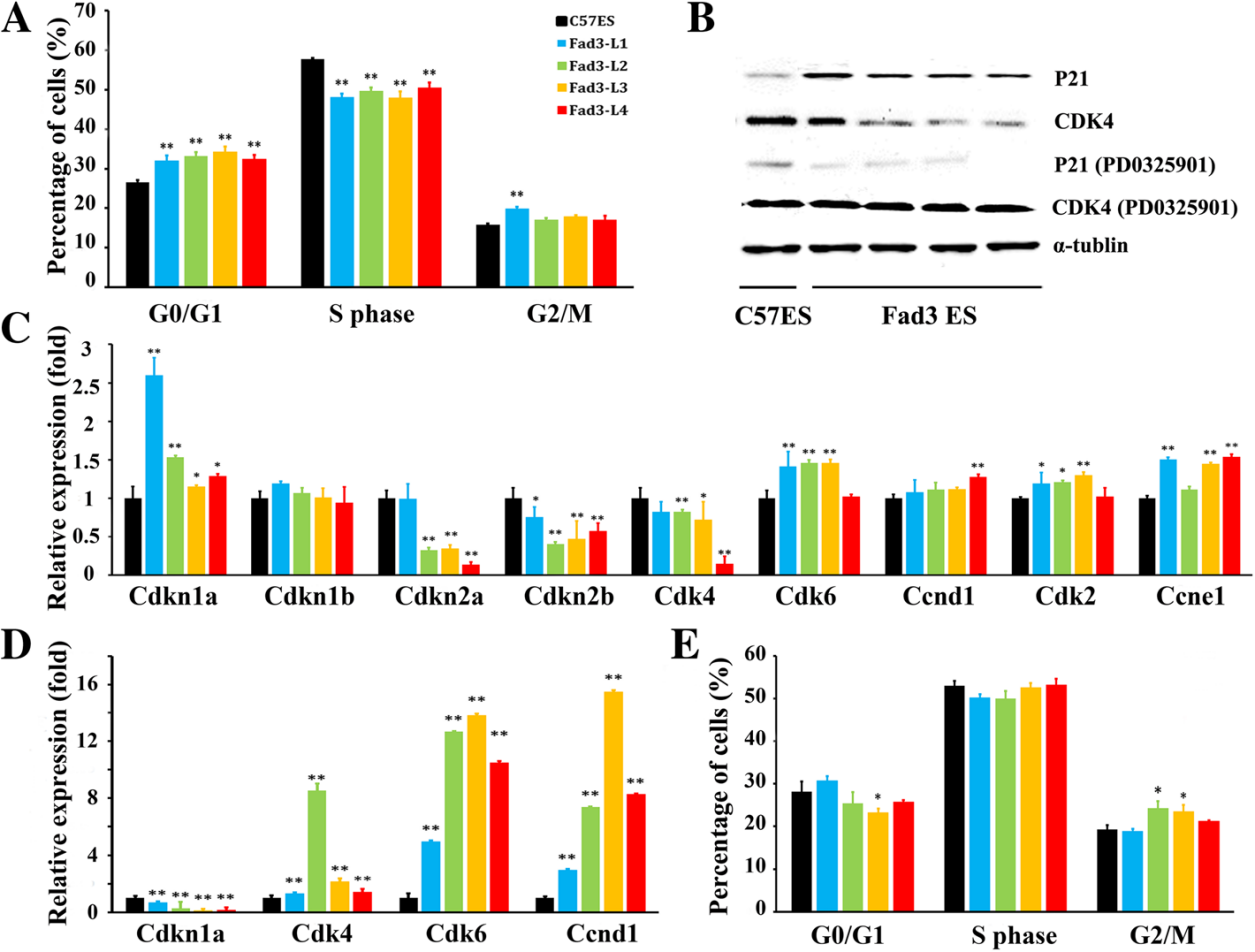
The expression of *fad3b* in ESCs caused G0/G1 arrest by regulating P21-Cdk4. **a**. Distribution of cell-cycle phases in *fad3b* ESCs and control cells. **b**. Western blot showing protein expression of cell-cycle-related proteins. **c**. The mRNA expression of cell-cycle-related genes in *fad3b* ESCs and control cells. **d**. The mRNA expression of cell-cycle-related genes after treatment with PD0325901 in *fad3b* ESCs and control cells. **e**. Distribution of cell-cycle phases after PD0325901 treatment in *fad3b* ESCs

To further study the prolonged cell cycle induced by *fad3b* expression, we next detected the cell-cycle distributions of *fad3b* ESCs. The results showed that 18 h after the 15th passage, only 26.57 ± 0.56% of the control cells were in the G0/G1 phase, whereas significantly more *fad3b* ESCs (L1: 32.05 ± 0.56%, L2: 33.27 ± 1.33%, L3: 34.24 ± 0.95%, and L4: 32.47 ± 1.05%) were in the G0-G1 phase (all *p* < 0.01) (Fig. 5a). At the same time, 57.67 ± 0.29% of the C57 ESCs were in S phase, which was significantly higher than that of the *fad3b* ESCs (L1: 48.06 ± 0.85%, L2: 49.60 ± 0.93%, L3: 47.89 ± 1.55%, and L4: 53.50 ± 1.29%; all p < 0.01). At 18 h after passaging, only the Fad3-L1 had more M phase cells than control cells (19.89 ± 0.48% vs. 15.76 ± 0.35%, p < 0.01), and there was no significant difference for the other *fad3b* ESC lines. From the cell-cycle analysis, we observed that the *fad3b* ESCs were arrested or prolonged in the G0/G1 phase, and this is perhaps the main reason for their growth slowdown.

According to the mRNA expression data, the expression of cyclin D1 in the *fad3b* ESCs was not significantly different from that of the controls (Fig. 5c). However, the expression of Cdk6, which has an important role during the G0-to-G1 transition [23], was significantly upregulated and that of Cdk4 was significantly down-regulated (Fig. 5c). The reduction of Cdk4 expression might elongate the G1 phase [24, 25]. The expression of Cdk2 and cyclin E was significantly upregulated, which has been associated with G1-to-S transition [26] (Fig. 5c).

Further analysis of the expression of checkpoint inhibition factors revealed that the expression of p21 (Cdkn1a) was significantly upregulated, the expression of p15 (Cdkn2b) and p16 (Cdkn2a) were significantly down-regulated, and the expression of p27 (Cdkn1b) did not change significantly (Fig. 5c). Downregulation of p15 and p16 does not inhibit the cell cycle [27]. The western blot results indicated that the protein expression of p21 in the *fad3b* ESCs was higher than that of the control cells and that of Cdk4 was lower (Fig. 5b). After addition of 1 μM PD0325901, an inhibitor of p21 expression, to the cell culture medium [28], the expression of p21 reduced, whereas the expression of Cdk4 increased (Fig. 5d and b), and the cell-cycle distributions of *fad3b* ESCs became similar to that of controls (Fig. 5e).

**Fig. 6:**
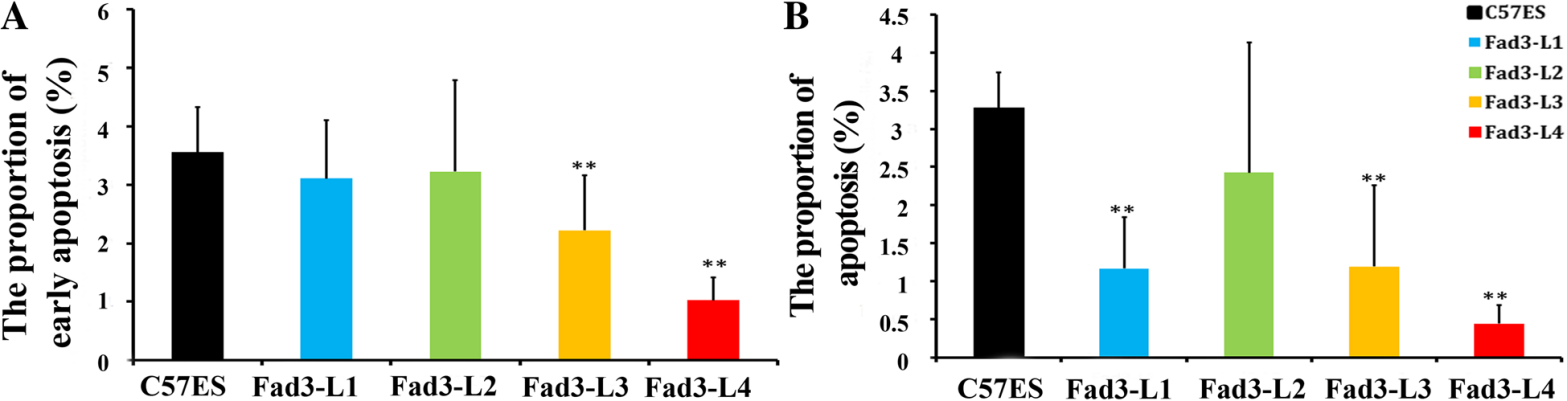
The expression of *fad3b* in ESCs had not effect on apoptosis. **a**. Early apoptosis frequency in *fad3b* ESCs and control cells. **b**. Apoptosis frequency in *fad3b* ESCs and control cells

Because apoptosis is also an important factor that affects cell proliferation, we analyzed apoptosis using flow cytometry. The proportion of cells in early apoptosis and late apoptosis in *fad3b* ESCs was slightly lower than that of control cells (Fig. 6a, b). Therefore, apoptosis was not a factor that affected the proliferation of *fad3b* ESCs.

These results show that the slowed cell proliferation in the *fad3b* ESCs was due to a prolonged G1 phase, which was probably due to p21-Cdk4, but not cyclin D-Cdk6 and cyclin E-Cdk2. Overall, our findings suggest that *fad3b* prolongs the cell cycle in the G1 phase but does not totally inhibit the G0-to-G1 or G1-to-S transition or apoptosis.

## Discussion

In this study, we obtained *fad3b*-expressing ESCs from blastocysts of *fad3b* transgenic mice and found no obvious differences in morphology or pluripotency between the *fad3b* ESCs and control C57 ESCs [29]. The expression of *fad3b* was stable in *fad3b* ESCs but was different between *fad3b* ESC lines. This difference may be caused by the copy numbers of *fad3b* integrated into the genome; the transgenic mice used in the experiment had three to four copies of *fad3b* [30]. Our analysis showed that the omega 3 PUFA content of the *fad3b* ESCs was significantly higher than that in controls. In addition, with prolonged in vitro culture time, the omega 6 to omega 3 ratios of the control cells increased, but there was no such change in the *fad3b* ESCs, indicating that the fatty-acid desaturation activity of *fad3b* is very stable in the mice ESCs.

The expression of *fad3b* significantly affected the proliferation of *fad3b* ESCs. This change was likely mediated by the effect of omega 3 PUFAs on the cell cycle and the subsequent arrest of cells in the G0/G1 phases. ESC proliferation and cell-cycle properties are different from those of somatic cells, in that ES cells have short G1 phases and a very long S phase [31]. The G1 phase of the ESC cell cycle has been correlated with lineage specifications [18, 32, 33]. However, studies by Gonzales et al. have shown that upregulation of differentiation markers was inhibited upon knockdown of S and G2 phase progression genes but not upon prolonging of G1 phase [18].

The restriction (R) point divides the G1 phase of the cell cycle into the early phase and late phase. The transition from early to late G1 is primarily regulated by the cyclin D-Cdk4/Cdk6 complexes, and the late G1 phase is characterized by cyclin E-Cdk2 complex activity [26]. In most cases, a prolonged G1 phase is associated with differentiation, but artificially extending the G1 phase by knocking down Cdk4/6 or overexpressing p21 did not significantly affect ESC pluripotency [18]. Mouse ESCs have constitutive cyclin E-Cdk2 activity [26, 34] and low level cyclin D-Cdk4/Cdk6 activity [17]. Cdk4 and Cdk6 have similar structure and functions in the G1 transition [35]. Cdk6 has an important role during the G0-to-G1 transition [23], and overexpression of cyclin D1 and Cdk4 shortens the G1 phase [24, 25]. A shorter G1 phase and increased G0-to-G1 transition is the functional secession between Cdk4 and Cdk6. We found that Cdk4 not Cdk6 is responsible for the elongation of G1 phase in *fad3b* ESCs.

Cdk inhibitors, including INK (which inhibits p15, p16, and p18) and CIP (which inhibits p21, p27, and p57), modulate the activity of Cdk complexes. We found that the increased p21 expression in our *fad3b* ESC lines was key for G0/G1 arrest. Notably, p21 inhibits cyclin D-Cdk4 activity via c-Jun N-terminal kinases (JNKs) [36–38]. Cdk4 activation is required for phosphorylation of the T172 of Cdk4 [39], a residue that is not present in Cdk6 and Cdk1/2. Thus, the binding of Cyclin D1-Cdk4 to p21could be activated by JNKs via S130 phosphorylation of p21 and T172 phosphorylation of Cdk4 [38]. In cancer cells and tissue stem cells, omega 3 PUFAs increased p21 expression to induce cell-cycle arrest and apoptosis or differentiation [11, 40]. However, we observed that *fad3b* ESCs have reduced apoptosis and remain in an undifferentiated state, indicating that the role of omega 3 PUFAs is different in ESCs. p21 is an important downstream target of the Akt2 [41], p53 [42], and Erk [43], and the specific pathway that regulates omega 3 and p21 is still debated.

In summary, we found that *fad3b* expression in mouse ESCs increased the level of omega 3 PUFAs without affecting ESC morphology and pluripotency. However, *fad3b* expression inhibited cell proliferation such that cells were arrested in the G0/G1 phase due to p21-induced reduction of Cdk4. Understanding the mechanisms of this effect could be helpful for developing new methods of creating pluripotent cells or for differentiating cells that are suitable for clinical applications.

## Conclusions

We found that expression of the *fad3b* gene in ESCs increased omega 3 PUFA content. Additionally, the proliferation of *fad3b*-expressing ESCs was inhibited, and a significantly higher proportion of *fad3b* ESCs were arrested in G0/G1 than control cells. The inhibition of cell proliferation was due to a prolonged G1 phase, but there was no inhibition of the G0-to-G1 transition or G1-to-S transition. The prolonged G1 phase in *fad3b* cells was probably induced by downregulation of Cdk4 expression via p21 upregulation. Therefore, omega 3 PUFAs may have unusual proliferative properties in undifferentiated ESCs.

## Additional file

Additional file 1: **Table S1.** The real time PCR primers. **Table S2.** The content of fatty acids in the cells. (DOCX 22 kb)

## Abbreviations

Ccnd1: G1/S-specific cyclin-D1
Ccne1: G1/S-specific cyclin-E1
Cdk2: Cyclin-dependent kinase 2
Cdk4: Cyclin-dependent kinase 4
Cdk6: Cyclin-dependent kinase 6
Cdkn1a: Cyclin-dependent kinase inhibitor 1A
Cdkn1b: Cyclin-dependent kinase inhibitor 1B
Cdkn2a: Cyclin-dependent kinase inhibitor 2A
Cdkn2b: Cyclin-dependent kinase inhibitor 2B
Cebpα: CCAAT/enhancer-binding protein alpha
DAPI: 4′,6-diamidino-2-phenylindole
DHA: Docosahexanoic acid
DPBS: Dulbecco’s phosphate-buffered saline
EPA: Eicosahexanoic acid
ESCs: Embryonic stem cells
Fabp4: Fatty acid-binding protein 4
JNKs: c-Jun N-terminal kinases
Lpl: Lipoprotein lipase
MUFAs: Monounsaturated fatty acids
PFA: Paraformaldehyde
Pnpla2: Patatin-like phospholipase domain-containing protein 2
Pparγ: Peroxisome proliferator-activated receptor γ
PUFAs: Polyunsaturated fatty acids
PVDF: Polyvinylidene fluoride
SDS: Sodium dodecyl sulfate
SFAs: Saturated fatty acids
TBST: Tris buffered saline with Tween
