# Supplementary material for: Omega 3 polyunsaturated fatty acids inhibit cell proliferation by regulating cell cycle in fad3b transgenic mouse embryonic stem cells
Source: Lipids Health Dis. 2018 Sep 8;17:210. doi: 10.1186/s12944-018-0862-x (PMC6129006; doi:10.1186/s12944-018-0862-x)
Supplement: Supplementary file 1 — Table S1. The real time PCR primers. Table S2. The content of fatty acids in the cells. (DOCX 22 kb) [file 12944_2018_862_MOESM1_ESM.docx]

**Supplementary table Legends**

Table S1. The real time PCR primers.

Table S2. The content of fatty acids in the cells.

Tab. S1.

| **PRIMERS** | | **SEQUENCES** |
| --- | --- | --- |
| Pparα-F | 5' –GGAGCCTAAGTTTGAGTTTGCTGTG-3’ | |
| Pparα-R | 5' –TGCAGCAGGTTGTCTTGGATG-3’ | |
| Lpl-F | 5' –CTCGCTCTCAGATGCCCTACAA-3’ | |
| Lpl-R | 5' –GTCCACCTCCGTGTAAATCAAGAA-3’ | |
| Fabp4-F | 5' –TGGGAACCTGGAAGCTTGTCTC-3’ | |
| Fabp4-R | 5' –GAATTCCACGCCCAGTTTGA-3’ | |
| Pnplα2-F | 5' –CTTGCCACTTTATGAGCTGAAGAAT-3’ | |
| Pnplα2-R | 5' –CGGTAGAGATTGCGAAGGTTG-3’ | |
| Cebpα-F | 5' –TTGAAGCACAATCGATCCATCC-3’ | |
| Cebpα-R | 5' –GCACACTGCCATTGCACAAG-3’ | |
| Fad3-F | 5’- ATGTTCTGGGCCGTCTTTGT-3’ | |
| Fad3-R | 5’- TTCTGGTGGTGGGTCTTGTG-3’ | |
| Cdk6-F | 5’- GGCGTACCCACAGAAACCATA-3’ | |
| Cdk6-R | 5’- AGGTAAGGGCCATCTGAAAACT-3’ | |
| Cdk4-F | 5’- ATGGCTGCCACTCGATATGAA-3’ | |
| Cdk4-R | 5’- TGCTCCTCCATTAGGAACTCTC-3’ | |
| Ccnd1-F | 5’- TGACTGCCGAGAAGTTGTGC-3’ | |
| Ccnd1-R | 5’- CTCATCCGCCTCTGGCATT-3’ | |
| Cdkn1a-F | 5’- CCTGGTGATGTCCGACCTG-3’ | |
| Cdkn1a-R | 5’- CCATGAGCGCATCGCAATC-3’ | |
| Cdkn1b-F | 5’- GGGCAGATACGAGTGGCAG-3’ | |
| Cdkn1b-R | 5’- TGAGACCCAATTAAAGGCACC-3’ | |
| Cdkn2b-F | 5’- CCCTGCCACCCTTACCAGA-3’ | |
| Cdkn2b-R | 5’- GCAGATACCTCGCAATGTCAC-3’ | |
| Cdkn2a-F | 5’- GCTCAACTACGGTGCAGATTC-3’ | |
| Cdkn2a-R | 5’- GCACGATGTCTTGATGTCCC-3’ | |
| Gapdh-F | 5’- TGGCCTTCCGTGTTCCTAC-3’ | |
| Gapdh-R | 5’- GAGTTGCTGTTGAAGTCGCA-3’ | |

Tab. S2

|  | **SFA (%)** | **MUFA (%)** | **PUFA (%)** | **ω-6 PUFA (%)** | **ω-3 PUFA (%)** | **ω-6/ω-3** |
| --- | --- | --- | --- | --- | --- | --- |
| C-P10 | 66.18±6.84 | 20.15±5.79 | 13.67±2.98 | 6.14±0.86 | 3.56±0.7 | 1.74±0.14 |
| C-P15 | 65.29±3.02 | 18.66±1.75 | 15.81±2.05 | 5.61±1.72 | 4.16±0.39 | 1.34±0.29 |
| C-P20 | 64.53±7.72 | 20.77±6.19 | 14.7±2.52 | 6.51±0.96 | 3.16±1.3 | 2.27±0.87 |
| C-P25 | 76.98±8.32 | 12.98±6.46 | 10.04±1.99 | 4.86±1.12 | 1.94±0.62 | 2.66±1.01 |
| C-P30 | 72.04±7.5 | 15.49±6.64 | 12.47±0.96 | 6.35±0.84 | 1.34±0.26 | 4.82±0.63 |
| C-Average | 69±5.35 | 17.61±3.3 | 13.34±2.22 | 5.89±0.67 | 2.83±1.17 | 2.56±1.36 |
|  |  |  |  |  |  |  |
| Fad3-L1-P10 | 69.98±8.21 | 17.91±5.2 | 12.1±3.02 | 4.04±0.25* | 5.08±0.9 | 0.81±0.17* |
| Fad3-L1-P15 | 62.98±2.87 | 15.22±0.56* | 21.8±2.65* | 6.97±0.41 | 7.45±0.97** | 0.95±0.14 |
| Fad3-L1-P20 | 64.43±3.46 | 18.77±1.24 | 16.8±4.54 | 6.29±2.6 | 6.52±2.05* | 0.95±0.14* |
| Fad3-L1-P25 | 63.16±8.99 | 20.17±8.59 | 16.68±1.76* | 6.15±0.49 | 5.39±0.48** | 1.15±0.15 |
| Fad3-L1-P30 | 67.04±5.35 | 17.36±1.93 | 15.6±3.93 | 6.08±2.11 | 5.1±1.85* | 1.21±0.17** |
| L1-Average | 65.52±2.98 | 17.89±1.83 | 16.6±3.48 | 5.9±1.1 | 5.91±1.04** | 1.01±0.16* |
|  |  |  |  |  |  |  |
| Fad3-L2-P10 | 57.88±8.7 | 24.05±4.19 | 18.07±4.57 | 5.88±1.08 | 7.08±1.44* | 0.84±0.13** |
| Fad3-L2-P15 | 53.8±5.19* | 26.27±3.11** | 19.93±2.26 | 6.23±0.26 | 8.3±3.03* | 0.78±0.27* |
| Fad3-L2-P20 | 55.21±5.83 | 23.97±2.47 | 20.82±3.36 | 6.45±1.05 | 9.68±2.15* | 0.67±0.05* |
| Fad3-L2-P25 | 58.87±1.98* | 22.21±2.21 | 19.35±1.74** | 5.42±1.25 | 7.83±0.23** | 0.69±0.17* |
| Fad3-L2-P30 | 56.19±3.42* | 22.36±1.58 | 21.45±1.86** | 6.03±1 | 9.72±1.42** | 0.64±0.19** |
| L2-Average | 56.39±2.03** | 23.77±1.64** | 19.92±1.31** | 6±0.39 | 8.52±1.16** | 0.73±0.08* |
|  |  |  |  |  |  |  |
| Fad3-L3-P10 | 58.4±2.99 | 21.08±2.38 | 20.52±2.73* | 5.99±1.99 | 10.05±0.63** | 0.6±0.22** |
| Fad3-L3-P15 | 51.1±1.79** | 29.79±2.36** | 19.11±0.71* | 6.64±0.26 | 8.39±0.37** | 0.79±0.07* |
| Fad3-L3-P20 | 56.98±1.19 | 22.94±1.85 | 20.08±2.82 | 5.86±0.72 | 9.64±1.13** | 0.61±0.01* |
| Fad3-L3-P25 | 51.67±9.99* | 25.94±10.36 | 22.4±3.82** | 7.85±0.9* | 9.46±0.05** | 0.83±0.1* |
| Fad3-L3-P30 | 51.48±9.77* | 25.32±3.95 | 23.19±6.31* | 6.12±0.82 | 10.22±1.09** | 0.6±0.1** |
| L3-Average | 53.93±3.48** | 25.02±3.3** | 21.06±1.69** | 6.49±0.82 | 9.55±0.72** | 0.69±0.11* |
|  |  |  |  |  |  |  |
| Fad3-L4-P10 | 62.84±8.33 | 21.37±4.65 | 15.23±6.72 | 3.63±1.76 | 7.1±6.02 | 0.57±0.23** |
| Fad3-L4-P15 | 67.71±9.91 | 20.82±5.98 | 18.14±1.01 | 4.12±0.6 | 8.53±1.3** | 0.48±0.01** |
| Fad3-L4-P20 | 68.72±3.73 | 14.89±2.48 | 16.87±0.97 | 4.05±0.48* | 7.81±0.95** | 0.53±0.12* |
| Fad3-L4-P25 | 63.94±1.79 | 19.3±1.51 | 16.75±2.32* | 4.84±0.65 | 7.19±1.96* | 0.69±0.09* |
| Fad3-L4-P30 | 66.85±3.41 | 17.1±3.48 | 16.05±1.99* | 5.61±1.08 | 5.89±0.25** | 0.95±0.16** |
| L4-Average | 66.01±2.51 | 18.7±2.7 | 16.61±1.08* | 4.45±0.78* | 7.3±0.98** | 0.64±0.19* |

Note: *:p<0.05;**:p<0.01.
